# Complete genome sequence of the halophile bacterium *Kushneria konosiri* X49^T^, isolated from salt-fermented *Konosirus punctatus*

**DOI:** 10.1186/s40793-018-0324-0

**Published:** 2018-10-01

**Authors:** Ji-Hyun Yun, Hojun Sung, Hyun Sik Kim, Euon Jung Tak, Woorim Kang, June-Young Lee, Dong-Wook Hyun, Pil Soo Kim, Jin-Woo Bae

**Affiliations:** 1 Department of Life and Nanopharmaceutical Sciences, Kyung Hee University, 1 Hoegi-dong, Dongdaemun-gu, Seoul, South Korea; 2 Department of Biology, Kyung Hee University, 1 Hoegi-dong, Dongdaemun-gu, Seoul, South Korea

**Keywords:** *Kushneria konosiri*, *Halomonadaceae*, Halophile, Complete genome, *Konosirus punctatus*

## Abstract

*Kushneria konosiri* X49^T^ is a member of the *Halomonadaceae* family within the order *Oceanospirillales* and can be isolated from salt-fermented larval gizzard shad. The genome of *K. konosiri* X49^T^ reported here provides a genetic basis for its halophilic character. Diverse genes were involved in salt-in and -out strategies enabling adaptation of X49^T^ to hypersaline environments. Due to resistance to high salt concentrations, genome research of *K. konosiri* X49^T^ will contribute to the improvement of environmental and biotechnological usage by enhancing understanding of the osmotic equilibrium in the cytoplasm. Its genome consists of 3,584,631 bp, with an average G + C content of 59.1%, and 3261 coding sequences, 12 rRNAs, 66 tRNAs, and 8 miscRNAs.

## Introduction

The gizzard shad, *Konosirus punctatus*, is a popular marine fish used as a food source in Northeast Asia and is usually consumed as a grilled dish, sushi, or jeotgal (or jeot). Jeotgal is a traditional Korean fermented food made by adding a substantial amount of solar salt to seafood such as fish, shrimp, or shellfish. During fermentation, jeotgal gains an extra flavor that may be caused by a microorganism derived from the environment, solar salt, or sea organisms. In an analysis of the microbiota of salt-fermented seafood, the strain X49^T^ (= KACC 14623^T^ = JCM 16805^T^) was isolated from the salt-fermented larval gizzard shad, known as Daemi-jeot in Korean [1]*.* Blast analysis and phylogenetic analysis using the 16S rRNA sequence revealed that the strain X49^T^ belongs to the genus *Kushneria*.

The genus *Kushneria* was first proposed as a novel genus by Sanchez-Porro in 2009, on the basis of phylogenetic analyses of 16S and 23S rRNA gene sequences [2]. It comprises a group of related Gram-negative, aerobic, motile, and rod- or oval-shaped bacteria. Most *Kushneria* strains have been isolated from saline environments and possess hypersaline resistance [2–7]. Strain X49^T^ also has a halophilic character [1]. At present, there are four sequenced *Kushneria* strains, but only the genome of *Kushneria marisflavi* KCCM 80003^T^ has been reported [8]. Thus, the genomic analysis of strain X49^T^ should help us to understand the genetic basis of adaptation to a hypersaline environment. The present study determined the classification and features of strain X49^T^, as well as its genome sequence and gene annotations.

## Organism information

### Classification and features

**Fig. 1:**
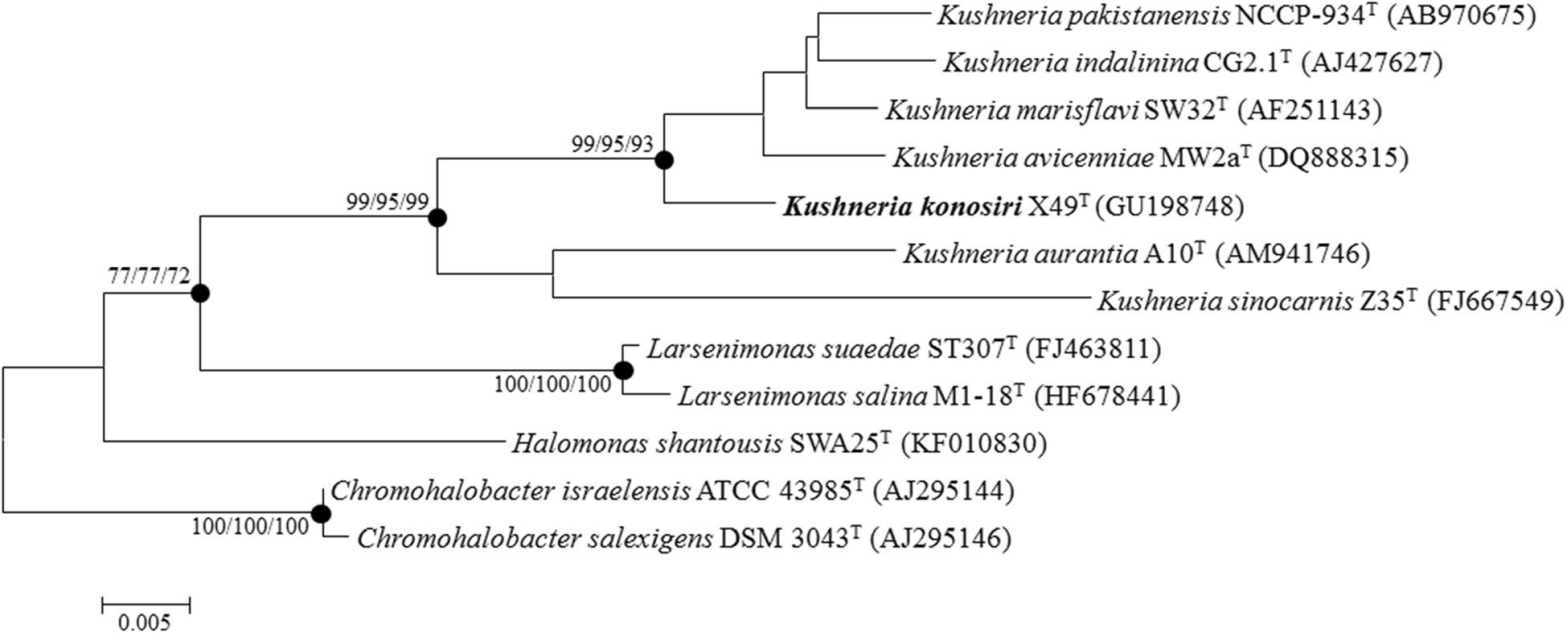
Neighbor-joining phylogenetic tree based on 16S rRNA gene sequences of *K. konosiri* X49^T^ and closely related taxa. Numbers at nodes indicate bootstrap values (over 70%, 1000 replicates) for neighbor-joining, maximum-likelihood, and maximum-parsimony. Closed circles indicate the nodes that were also generated by maximum-likelihood and maximum-parsimony. Scale bar, 0.005 accumulated changes per nucleotide

**Fig. 2:**
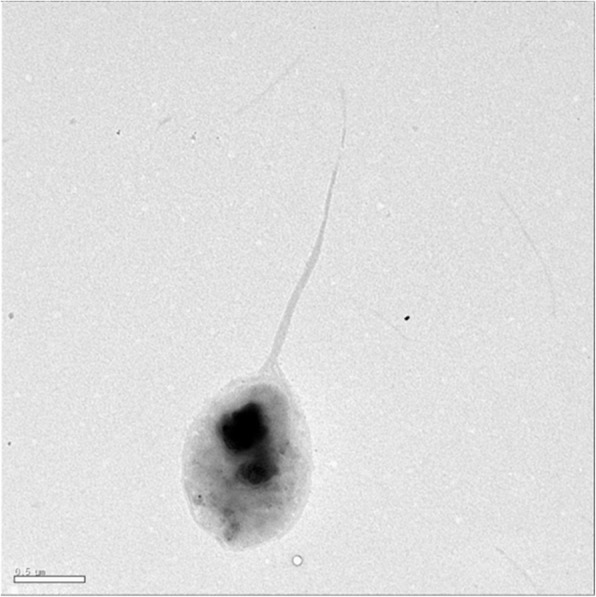
Transmission electron micrograph (TEM) of *K. konosiri* X49^T^. The TEM (JEM-1010; JEOL) image was obtained from a previous study [1]

Serially diluted suspensions of Daemi-jeot were plated directly on Marine agar medium and maintained under aerobic condition at 25 ± 1 °C for 14 days. To obtain pure isolates, a single colony was repeatedly transferred to new agar plates. Comparison between the 16S rRNA gene sequence of strain X49^T^ (Accession number: GU198748) and those obtained using NCBI BLASTN [9] with the settings for highly similar sequences produced 100 hits: 47, 36, 1, and 1 from the genera *Kushneria*, *Halomonas*, *Halomonadaceae*, and *Chromohalobacter*, respectively, and the remaining 15 from uncultured bacteria. The validated species with the maximum sequence similarity was *K. marisflavi* SW32^T^ (NR_025094), which shared a sequence identity of 98.63%. Phylogenetic analysis using MEGA6 [10] based on 16S rRNA gene sequences of the *Kushneria* members and related taxa showed that strain X49^T^ was within the cluster comprising the genus *Kushneria* (Fig. 1). Strain X49^T^ is classified as *Proteobacteria*, *Oceanospirillales*, *Halomonadaceae*, and *Kushneria*, and is named *Kushneria konosiri*. Characteristics of *K. konosiri* X49^T^ are presented in Table 1. The cells were aerobic, Gram-negative, rod- or oval-shaped, and 1.2–3.2 μm in length and 0.5–1.0 μm in width. A flagellum was observed (Fig. 2). The colonies were orange-colored and circular with entire margins on marine agar medium. Growth was observed at 10–37 °C, at pH 4.5–8.5, and in the presence of 0–26% (*w*/*v*) NaCl. The physiological characteristics, such as the growth substrates of *K. konosiri* X49^T^, have been described in detail previously [1].

**Table 1:** Classification and general features of *K. konosiri* X49^T^ according to the Minimum Information about a Genome Sequence (MIGS) recommendations

MIGS ID	Property	Term	Evidence code^a^
	Classification	Domain *Bacteria*	TAS [28]
		Phylum *Proteobacteria*	TAS [29]
		Class *Gammaproteobacteria*	TAS [30]
		Order *Oceanospirillales*	TAS [31]
		Family *Halomonadaceae*	TAS [32]
		Genus *Kushneria*	TAS [2]
		Species *Kushneria konosiri*	TAS [1]
		Type strain X49^T^ (Accession GU198748)	TAS [1]
	Gram stain	Negative	TAS [1]
	Cell shape	Rod, oval-shaped	TAS [1]
	Motility	Motile	TAS [1]
	Sporulation	Not reported	TAS [1]
	Temperature range	10–37 °C	TAS [1]
	Optimum temperature	15–25 °C	TAS [1]
	pH range	pH 4.5–8.5	TAS [1]
	Optimum pH range	pH 5.0–7.0	TAS [1]
	Carbon source	Heterotroph	TAS [1]
MIGS-6	Habitat	Fermented food	TAS [1]
MIGS-6.3	Salinity	0–26% NaCl (*w*/*v*)	TAS [1]
MIGS-22	Oxygen requirement	Aerobic	TAS [1]
MIGS-15	Biotic relationship	Free-living	NAS
MIGS-14	Pathogenicity	Not reported	
MIGS-4	Geographic isolation	South Korea: Goheung	TAS [1]
MIGS-5	Sample collection date	Apr-09	
MIGS-4.1	Latitude	Not reported	
MIGS-4.1	Longitude	Not reported	
MIGS-4.3	Depth	Not reported	
MIGS-4.4	Altitude	Not reported	

The evidence codes are as follows. TAS: traceable author statement (i.e., a direct report exists in the literature). NAS: non-traceable author statement (i.e., not observed directly in a living, isolated sample, but based on a generally accepted property of the species, or anecdotal evidence). These evidence codes are from the Gene Ontology project [13]

#### Chemotaxonomic data

The predominant cellular fatty acids (> 10% of the total) in *K. konosiri* X49^T^ were C_16:0_, C_18:1_ *ω*7*c*, Summed feature 3 (C_16:1_ *ω*7*c* and/or C_16:1_ *ω*6*c*), and C_12:0_ 3OH. The respiratory quinone was ubiquinone Q9, and minor quinones were ubiquinone Q8 and Q10. The polar lipids contained diphosphatidylglycerol, phosphatidylglycerol, phosphatidylethanolamine, phosphatidylserine, two unidentified aminophospholipids, two unidentified phospholipids, and two unidentified lipids.

## Genome sequencing information

### Genome project history

*K. konosiri* X49^T^ was selected for genome sequencing based on its environmental potential and this genome sequencing was part of the Agricultural Microbiome R&D Program (grant number: 914006–4) at the Korea Institute of Planning and Evaluation for Technology in Food, Agriculture, Forestry (IPET) funded by the Ministry of Agriculture, Food and Rural Affairs (MAFRA). The genome sequence was deposited in DDBJ/EMBL/GenBank under accession number CP021323, and the genome project was deposited in the GOLD [11] under Gp0223024. The sequencing and annotation were performed by Macrogen (Seoul, Korea). The details of the project information and the associations with MIGS [12] are shown in Table 2.

**Table 2:** Genome sequencing project information

MIGS ID	Property	Term
MIGS-31	Finishing quality	Finished
MIGS-28	Libraries used	20 kb SMRTbell library
MIGS-28.2	Number of reads	160,304 sequencing reads
MIGS-29	Sequencing platforms	PacBio RSII platform
MIGS-31.2	Fold coverage	× 224
MIGS-30	Assemblers	HGAP v.3
MIGS-32	Gene calling method	IMG annotation pipeline v.4.15.1.
	Locus Tag	B9G99
	GenBank ID	CP021323
	GenBank Date of Release	2017-05-24
	GOLD ID	Gp0223024
	BIOPROJECT	PRJNA383456
MIGS-13	Source material identifier	KACC 14623^T^, JCM 16805^T^
	Project relevance	Environmental

### Growth conditions and genomic DNA preparation

*K. konosiri* X49^T^ (lab stored, = KACC 14623^T^ = JCM 16805^T^) was cultured aerobically in LB broth (BD, USA) containing NaCl (4% w/v) at 30 ± 1 °C for 3 days. The genomic DNA of *K. konosiri* X49^T^ was extracted using a MG™ Genomic DNA Purification kit (Macrogen, Korea) according to the manufacturer’s instructions.

### Genome sequencing and assembly

For library preparation, gDNA was sheared with g-TUBE (Covaris Inc., USA) and then used for library preparation by ligating SMRTbell adaptors (20 kb SMRTbell library). The sequences of the generated library were sequenced using the PacBio RSII system, SMRT sequencing with DNA Sequencing Reagent Kit P6 and SMRT Cells 8Pac V3 (Pacific Biosciences). The sequencing generated 1,199,776,790 bp with 160,304 reads. After filtering of sequences that were shorter than 50 bp, 1,199,771,927 bp sequences with 160,189 subreads remained. Assembly was performed using software RS HGAP v3, which consists of pre-assembly, de novo assembly with Celera® Assembler, and assembly polishing with Quiver. Assembly resulted in one scaffold with the complete genome in circular form and 244-fold coverage.

### Genome annotation

**Fig. 3:**
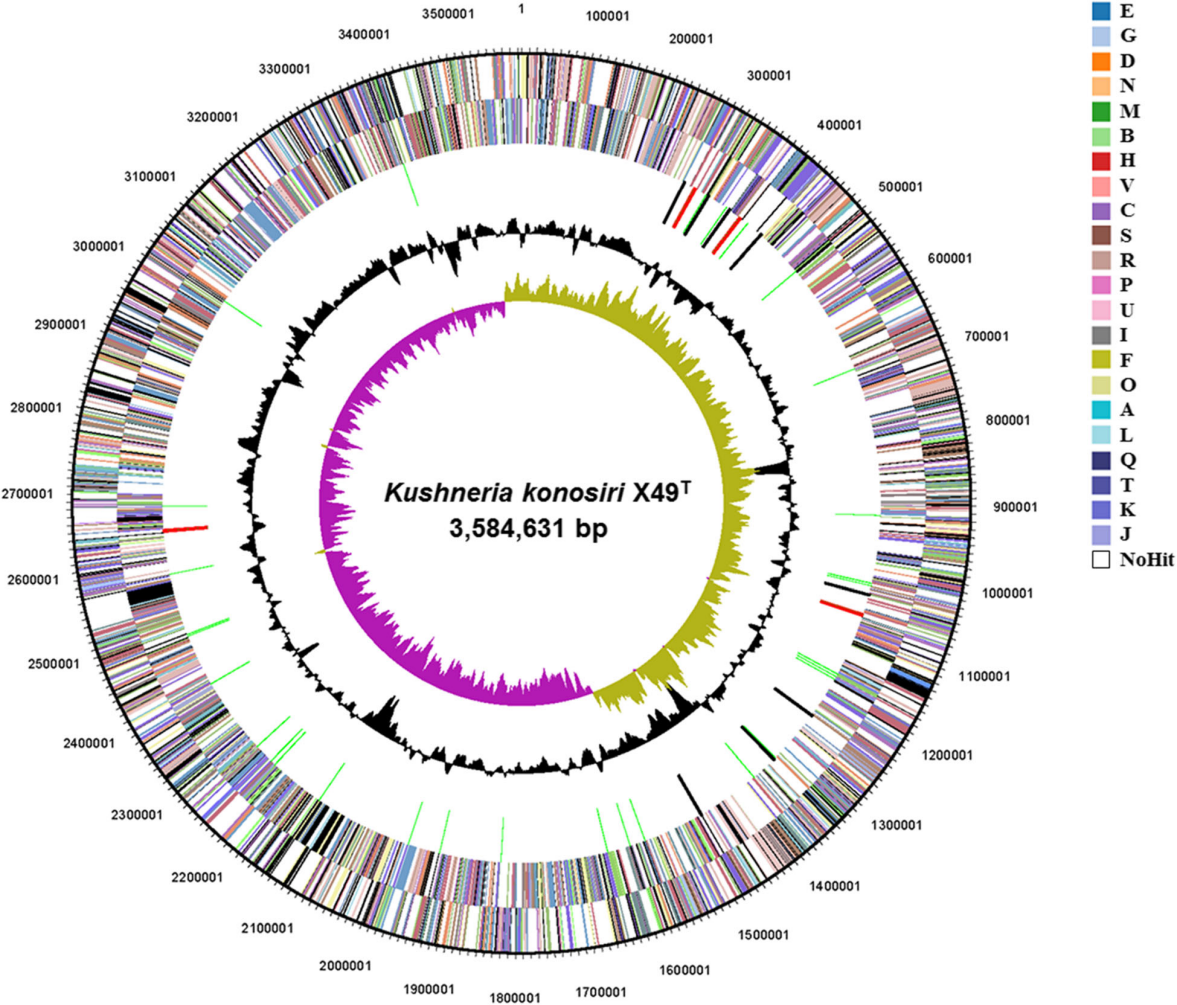
Circular map of the complete *K. konosiri* X49^T^ genome. Marked characteristics are shown from the outside to the center: the number of bases, COG on forward strand, COG on reverse strand, RNA genes (tRNAs, green bars; rRNAs, red bars; other RNAs, black bars), GC content, and GC skew. Individual genes are colored according to COG categories

Annotation of the assembled genome was performed using the DOE-JGI Microbial Genome Annotation Pipeline v.4.15.1 [13]. The gene prediction was carried out using the IMG-ER platform. Comparisons of the predicted ORFs using the KEGG [14], NCBI COG [15], Pfam [16], TIGRfam [17], and InterPro [18] databases were conducted during gene annotation. Additional gene prediction analyses and functional assignment were carried out using the NCBI PGAP [19] and the RAST with the gene caller classicRAST [20] based on the SEED [21]. CRISPR system analysis was carried out using the web-based interface CRISPRFinder (http://crispr.i2bc.paris-saclay.fr/). The chromosome map of *K. konosiri* X49^T^ was obtained from the output of the IMG pipeline (Fig. 3).

## Genome properties

The genome of *K. konosiri* X49^T^ comprised a single circular chromosome with a length of 3,584,631 bp and a G + C content of 59.1% (Fig. 3 and Table 3). Of the 3347 predicted genes, 3261 were protein-coding. According to tRNAscan-SE and RNAmmer 1.2, 66 tRNA, 12 rRNA (four 5S rRNA, four 16S rRNA, and four 23S rRNA genes), and 8 miscRNA genes were found in the genome. The genome contained one CRISPR structure with 25 spacers of 28 bp and two putative CRISPRs. The number of CDSs including signal peptides was 239 (7.14%). The majority of the protein-coding genes (2815 genes; 84.1%) were assigned to functional categories, while the remainder were annotated as hypothetical proteins (446 genes). The properties and statistics of the genome are summarized in Table 3, and the distributions of genes among the functional categories of COG are shown in Table 4.

**Table 3:** Genome statistics

Attribute	Value	% of Total
Genome size (bp)	3,584,631	100%
DNA coding (bp)	3,211,842	89.60%
DNA G + C (bp)	2,118,687	59.10%
DNA scaffolds	1	100%
Total genes	3347	100%
Protein coding genes	3261	97.43%
RNA genes	86	2.57%
Pseudo genes	73	2.18%
Genes in internal clusters	611	18.26%
Genes with function prediction	2815	84.11%
Genes assigned to COGs	2568	76.73
Genes with Pfam domains	2913	87.03%
Genes with signal peptides	239	7.14%
Genes with transmembrane helices	823	24.59%
CRISPR repeats	1	0

**Table 4:** Number of genes associated with general COGs functional categories

Code	Value	%age	Description
J	222	6.81%	Translation, ribosomal structure and biogenesis
A	1	0.03%	RNA processing and modification
K	179	5.49%	Transcription
L	112	3.43%	Replication, recombination and repair
B	1	0.03%	Chromatin structure and dynamics
D	32	0.98%	Cell cycle control, cell division, chromosome partitioning
V	54	1.66%	Defense mechanisms
T	134	4.11%	Signal transduction mechanisms
M	195	5.98%	Cell wall/membrane/envelope biogenesis
N	80	2.45%	Cell motility
U	35	1.07%	Intracellular trafficking, secretion, and vesicular transport
O	108	3.31%	Posttranslational modification, protein turnover, chaperones
C	197	6.04%	Energy production and conversion
G	216	6.62%	Carbohydrate transport and metabolism
E	264	8.10%	Amino acid transport and metabolism
F	84	2.58%	Nucleotide transport and metabolism
H	179	5.49%	Coenzyme transport and metabolism
I	108	3.31%	Lipid transport and metabolism
P	174	5.34%	Inorganic ion transport and metabolism
Q	73	2.24%	Secondary metabolites biosynthesis, transport and catabolism
R	268	8.22%	General function prediction only
S	138	4.23%	Function unknown
–	779	23.89%	Not in COGs

The total is based on the total number of protein coding genes (3261) in the genome

## Insights from the genome sequence

### Comparative genomics

To determine the genomic relatedness between *K. konosiri* X49^T^ and closest relative strain *K. marisflavi* SW32^T^, the ANI value was calculated using an online calculator (https://www.ezbiocloud.net/tools/ani). The ANI value between two whole genome sequences was 89.32%. This value was well below the threshold of 95%; this suggested that two strains represent genotypically distinct species.

### Hypersaline adaptation

**Fig. 4:**
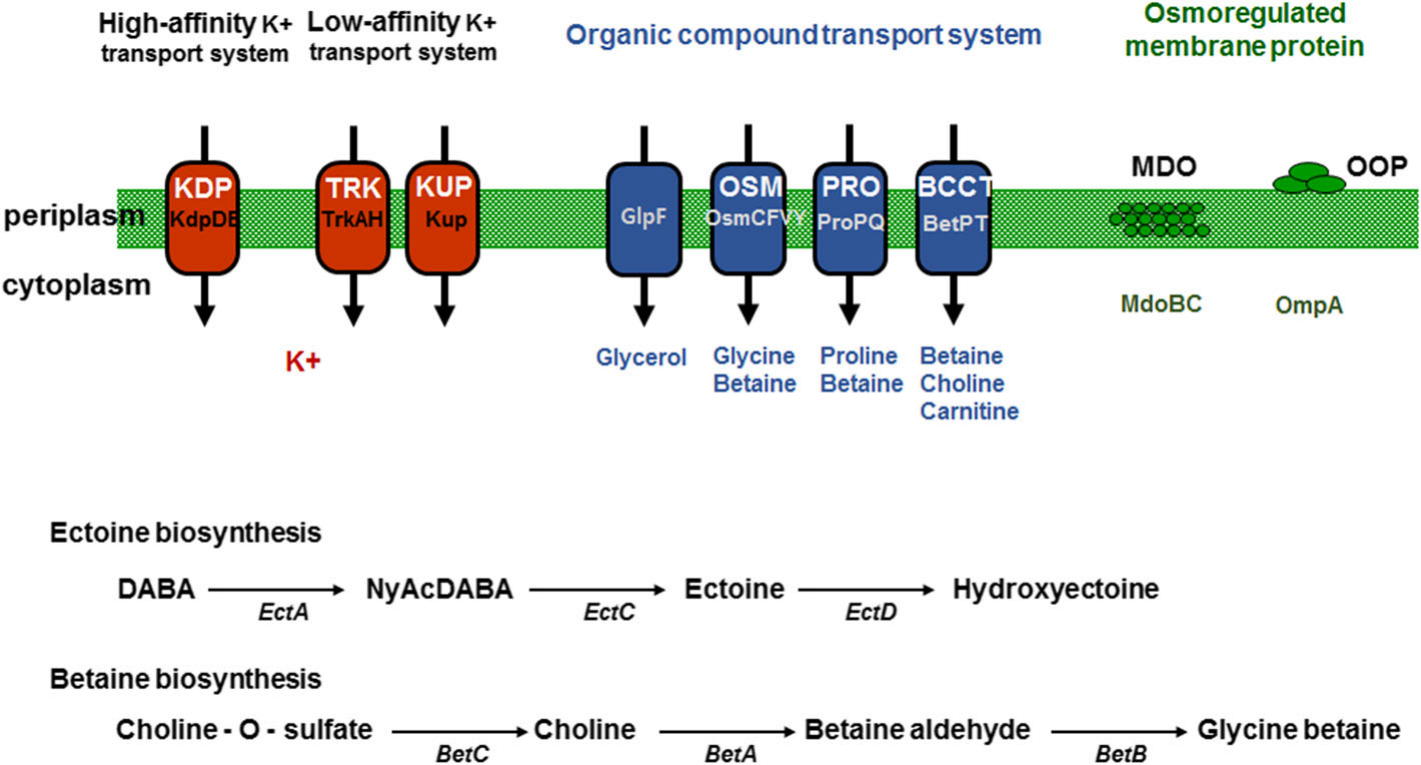
Genomic prediction of the osmoregulation in *K. konosiri* X49^T^. Trk, K^+^ transport; KUP, K^+^ uptake permease; Kdp, K^+^-dependent ATPase; BCCT, betaine-carnitine-choline transport; Osm, osmotically inducible protein; MDO, membrane-derived oligosaccharides; OOP, OmpA-OmpF Porin; EctA, L-2,4-diaminobutyric acid acetyltransferase; EctC, L-ectoine synthase; EctD, ectoine hydrolase; BetA, choline dehydrogenase; BetB, betaine aldehyde dehydrogenase; BetC, choline sulfatase protein

*Kushneria* members possess hypersaline resistance and *K. konosiri* X49^T^ can grow optimally at 11–19% (*w*/*v*) NaCl and survive in the presence of 26% (w/v) NaCl [1]. To balance the osmotic pressure between the inside and outside of the cell in the hypersaline habitat, theses halophile microorganisms increase the internal osmolarity of the cytoplasm using inorganic ions (mostly potassium ions) or organic compounds (mainly ectoine, choline, glycine betaine, and proline betaine, etc.), which are known as compatible solutes for the exclusion of salt ions. Genome analysis through the IMG pipeline revealed that *K. konosiri* X49^T^ can adapt its salt tolerance using several functional genes (Fig. 4). In response to changes in the external osmolality, potassium is accumulated in the cytoplasm by transport via the Trk-type transport systems, KUP system, or Kdp two-component system [22–24]. With increasing potassium levels, osmoprotective compounds are accumulated by synthesis and/or uptake in the cytoplasm as a bi-phasic response [22]. Also, expression of membrane proteins such as OMPs and of MDOs is affected by environmental osmolarity [25, 26].

To regulate the osmolarity between cytoplasm and environment, the genome of *K. konosiri* X49^T^ encodes potassium uptake-related loci: the Trk system (low-affinity potassium transport; two TrkA and two TrkH genes), KUP system (two potassium uptake permeases), and Kdp two-component system (one sensor histidine kinase KdpD and one operon response regulator KdpE). The X49^T^ genome also encodes a variety of organic compound regulation systems including ectoine biosynthesis-related genes (one EctA, L-2,4-diaminobutyric acid acetyltransferase; one EctC, L-ectoine synthase; and two EctD, ectoine hydrolases), betaine uptake-related genes (ten members of the betaine-carnitine-choline transport family; two BetPT, proline-betaine transporters; and six OsmC, F, V, and Y, glycine-betaine transporters), betaine biosynthesis-related genes (one BetA, choline dehydrogenase; two BetB, betaine aldehyde dehydrogenases; and one BetC, choline sulfatase), and one glycerol uptake facilitator protein. The osmoregulated membrane protein-related genes (three OmpA-OmpF Porin Family and osmoregulated periplasmic glucan biosynthesis) and osmoregulated periplasmic glucan-related genes (two MdoC, glucan biosynthesis proteins; one MdoB, phosphoglycerol transferase MdoB-like AlkP superfamily enzyme) were also found in the genome (Fig. 4).

### Carotenoid biosynthesis

**Fig. 5:**
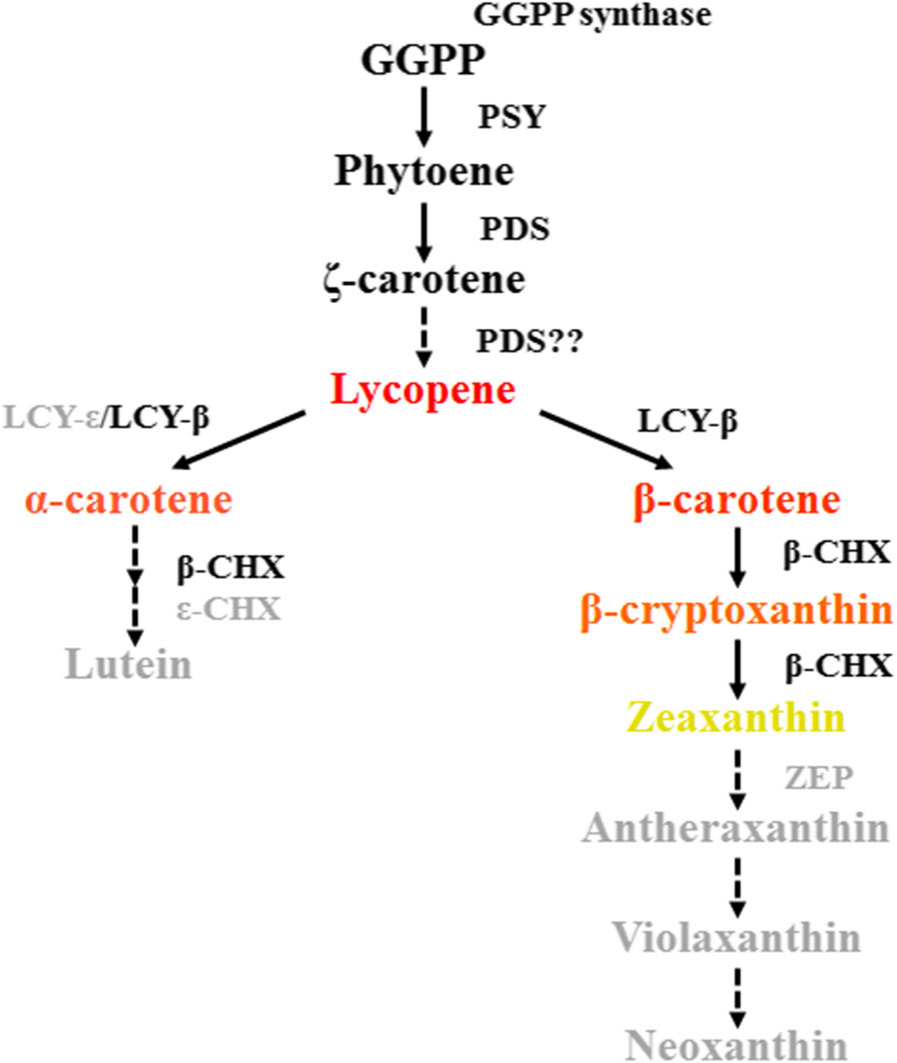
Carotenoid biosynthesis pathway in *K. konosiri* X49^T^. GGPP synthase, geranylgeranyl diphosphate synthase; PSY, phytoene synthase; PDS, phytoene desaturase; LCY-β, lycopene beta-cyclase; LCY-ε, lycopene ε-cyclase; β-CHX, beta-carotene 3-hydroxylase; ε-CHX, ε-carotene 3-hydroxylase. The font in gray color represents missing enzyme-coding genes and unproduced pigment in *K. konosiri* X49^T^

Carotenoids are naturally occurring pigments that not only act as antioxidants but also enhance salt stress tolerance [27]. The first step of carotenoid biosynthesis starts by formation of one phytoene from two molecules of GGPP, and phytoene is converted to α- or β-carotene along the biosynthesis pathway. α- or β-carotene is transformed into xanthophyll by obtaining an oxygen atom (Fig. 5). The colonies of *K. konosiri* X49^T^ were orange. The genome of *K. konosiri* X49^T^ encodes genes related to carotenoid biosynthesis: two GGPP synthases, one phytoene synthase, two phytoene desaturases, one lycopene β-cyclase, one β-carotene hydroxylase, and one enhancing lycopene biosynthesis protein 2. However, there is no xanthophyll cycle-related gene, ZEP, which is key for conversion of zeaxanthin to violaxanthin, suggesting that the orange color of the colony of *K. konosiri* is derived from β-carotene or β-cryptoxanthin. These carotenoids may act also as the potential osmoprotectants (Fig. 5).

## Conclusions

The orange pigmented *K. konosiri* X49^T^ was isolated from a salt-fermented food, Daemi-jeot, and was resistant to a hypersaline environment. Whole genome sequence analysis and physiological observations leads us to conclude that *K. konosiri* X49^T^ is a orange-coloured halophile and its capabilities of a cellular response are enabled by a variety of genes determining the ‘carotenoid biosynthesis’ and ‘inorganic or organic transport and metabolism’. The presence of ectoine and betaine biosynthesis genes or transport system related genes demonstrates the possibility of a cellular response to high osmolarity through biosynthesis of ectoine and betaine to protect the cell from stress. As ectoine or betaine can play as a protectant under stress condition, the genome sequence of *K. konosiri* X49^T^ may provide the molecular basis for its hypersaline tolerance and may lead to new development in its diverse biotechnological applications comprising environmental, medical and biofuel industries.

## Abbreviations

ANI: Average-nucleotide identity
COG: Clusters of orthologous groups
CRISPR: Clustered regularly interspaced short palindromic repeat
DOE: Department of energy
GGPP: Geranylgeranyl diphosphate
*GOLD*: Genomes on line database
JGI: Joint genome Institute
KEGG: Kyoto encyclopedia of genes and genomes
LB: Luria–bertani
MIGS: Minimum information about a genome sequence
MOD: Membrane-derived oligosaccharides
OMP: Outer membrane proteins
PGAP: Prokaryotic genome annotation pipeline
RAST: Rapid annotation using subsystem technology
SMRT: Single-molecule real-time
ZEP: Zeaxanthin epoxidase
